# Latent Tuberculosis in Psoriasis Patients on Biologic Therapies: Real-World Data from a Care Center in Romania

**DOI:** 10.3390/medicina59061015

**Published:** 2023-05-24

**Authors:** Doriana-Sorina Lupea-Chilom, Caius Silviu Solovan, Simona Sorina Farcas, Armand Gogulescu, Nicoleta Ioana Andreescu

**Affiliations:** 1 Department of Dermatology, University of Medicine and Pharmacy “Victor Babeş”, Eftimie Murgu Sq. No. 2, 300041 Timisoara, Romania; doriana.chilom@umft.ro (D.-S.L.-C.); caius.solovan@gmail.com (C.S.S.); 2 Department of Microscopic Morphology—Genetics, Center of Genomic Medicine, University of Medicine and Pharmacy “Victor Babes”, Eftimie Murgu Sq. No. 2, 300041 Timisoara, Romania; andreescu.nicoleta@umft.ro; 3 Department of Medical Rehabilitation, University of Medicine and Pharmacy “Victor Babeş”, Eftimie Murgu Sq. No. 2, 300041 Timisoara, Romania; gogulescu.armand@umft.ro

**Keywords:** psoriasis, tuberculosis, biologics, methotrexate

## Abstract

*Background and Objectives:* Psoriasis is a chronic and inflammatory condition that has a huge impact on the patient’s quality of life. Biological treatment improved psoriasis therapy, with impressive results seen in the evolution of the disease and the patient’s quality of life. However, the risk of mycobacterium tuberculosis (MTB) infection reactivation is well-known to biological therapy, which raises problems especially in an endemic country. *Materials and Methods:* In this study, we followed moderate to severe psoriasis patients who had latent tuberculosis infection (LTBI) following treatment with a biological therapy approved in Romania. *Results:* The patients were evaluated at baseline and then followed-up with Mantoux tests and chest X-rays every year, resulting in 54 patients being diagnosed with LTBI. At the initial evaluation, 30 patients with LTBI were identified, and 24 more were identified during biological therapy. These patients were given prophylactic treatment. Out of the 97 participants in this retrospective study, 25 required association of methotrexate (MTX) alongside biological therapy. We compared the prevalence of positive Mantoux tests in patients with combined therapy with that of patients only on biological treatment, and the results were higher in the combined therapy group. *Conclusion*: All the patients in the study were vaccinated against tuberculosis (TB) after birth, and none were diagnosed with active tuberculosis (aTB) before or after the start of therapy according to the pulmonologist.

## 1. Introduction

About 2% of the world’s population is affected by psoriasis, which is a chronic disease whose treatment is continuously improving, bringing patients increasingly effective therapeutic options that result in long-lasting remissions [1]. Evidence on the incidence and prevalence of psoriasis is increasing which leads to a growing interest in the substrate of this disease, its effects on the entire body, and effective and safe therapies [2]. It is a debilitating condition due to the physical and mental discomfort it produces [3]. Biological agents have greatly improved the response to treatment and the quality of life for psoriatic patients. A cross-sectional observational study in Japan, published by Okubo Y. et al., showed statistically important differences when looking at those on biologic therapy versus those without. Biologic users had significantly lower Dermatology Life Quality Index (DLQI) scores and greater degree of satisfaction [4]. Even if biological agents have improved therapeutic safety compared to conventional systemic therapies [5], there are still various therapeutic risks. Although this therapy has been available to psoriatic patients for many years, demonstrating its positive effects on skin and arthropathic lesions, there are still no firmly established criteria in choosing the molecule adapted to patients. This is difficult to achieve given the continuous research needed to discover targeted therapies with better efficiency, efficacy and safety profile. The most common comorbidities that coexist with psoriasis and the appearance of infections are criteria that give direction in the choice of therapy for plaque psoriasis. According to the National Therapeutic Protocol for psoriasis treatment approved in 2022, the following biological agents are available in Romania: inhibitors of tumor necrosis factor (TNF)-α including etanercept, certolizumab pegol, adalimumb, infliximab; interleukin (IL)-17, inhibitors such as ixekizumab and secukinumab; anti IL-12/23 agents such as ustekinumab; IL-23 inhibitors such as guselkumab, risankizumab and tildrakizumab.

Biological treatment also includes risks of infections related to the decreased immunity of patients. Common infections, which occur in the entire population, can be more frequent in biological users and can have greater severity, which necessitates hospitalization or endangers the patient’s life. Common infections include upper airway infections, urinary tract infections, mycotic infections. The most common infections reported as a result of biological therapy are mild to moderate infections, which do not require stopping the anti-psoriatic therapy [6].

Tuberculous infection (TBI) is a condition that requires increased attention given the negative effects it has on patients but also for the community. The increased risk of reactivation of TB requires an assessment to detect aTB or LTBI before starting biological treatment. It is repeated every 12 months for those who did not have chemoprophylaxis during this period. Romania is one of the countries with a high rate of MTB infection (59.6/100,000 inhabitants in 2019) [7], so it is necessary to pay more attention to those at greater risk of TB, including patients with biological treatment.

In this observational retrospective study, we followed patients with moderate or severe psoriasis who were treated with biological agents and the relationship between treatment and the appearance of aTB or LTBI.

## 2. Materials and Methods

We performed a retrospective observational study at the University Clinic of Dermatology and Venereology Timișoara. Parameters included patients over 18 years old, diagnosed with severe plaque psoriasis, based on clinical signs and symptoms and histopathological examination, who were treated with biological therapy (inhibitors of TNF-α, IL-12/IL-23, IL-17, IL-23). The patients were evaluated at the time of initiating the treatment through a Mantoux test and pulmonary X-ray and followed up for a mean period of 6.9 years after initiation (min = 1 year, max = 17 years). During this time, the patients performed periodic evaluations every 12 months (±1 month) by Mantoux test and chest X-ray as recommended by the Romanian National Protocol Guide for the use of biological treatments in moderate to severe psoriasis. A Bacillus Calmette–Guérin (BCG) vaccination after birth was recorded for all participants. For initial and periodic evaluation of the patients, the area affected and severity index score (PASI score), blood tests (complete blood count, blood biochemistry, tests for hepatitis B and C, HIV tests and bacterial or fungal infections tests) were also performed. The Mantoux test was performed with 5 units of purified protein derivative (PPD) and 1 mL serum injected intradermally on the ventral forearm. The tuberculin intradermal reaction (IDR) was evaluated 72 h after injection and test was considered positive if there was induration equal or greater than 10 mm as measured along the longest axis. Patients with a positive Mantoux test but without active lesions on the chest X-ray and no symptoms for TBI, were diagnosed with LTBI.

Data was collected in an excel spreadsheet and descriptive statistics included mean values, standard deviation (SD), standard error (SE) of the continuous variables and percentages of the categorical variables. Chi-square analyses and *t*-tests were performed where appropriate. A *p* value of less than 0.05 was considered statistically significant.

## 3. Results

Based on the inclusion criteria, a total of 97 patients (39 females and 58 males) were identified. All patients were diagnosed both clinically and through histological analysis with plaque psoriasis vulgaris. Some of the patients also had psoriasis of special areas, such as involvement of the scalp (34%), genital area (2%), and nails (5%). The association of two special areas was observed in 5% of patients for both the scalp and genitals, and 10% for both the scalp and nails. The most common associated comorbidities have been reported in Table 1.

Patients were initiated into treatment with the following biological molecules: 75 were treated with TNF-α inhibitors (34 adalimumab, 24 etanercept, 17 infliximab), seven patients had therapy with anti IL-17 (five ixekizumab, two secukinumab), six patients were on anti-IL-23 (three guselkumab, three risankizumab), and nine of them with anti-IL-12/IL-23 (ustekinumab). The average age of the patients included in the study is 57.7 years old. The mean of the time between the moment of establishing the diagnosis of psoriasis until the start of the biological treatment was 18.3 years.

During systematic evaluations performed to monitor the efficiency and efficacy of the psoriatic treatment, data were collected about the different infections occurred during the biological therapy. Gastrointestinal infections, pharyngeal infections, urinary infections, candidiasis, LTBI, pneumonia, and viral hepatitis with hepatitis B and C virus were recorded. Fifty (50)% of those who had infections developed two or more infections during biological therapy. None of them required discontinuation of psoriatic treatment to cure the infections, nor did they have any major complications as a result of these infections. The frequency of these infections can be seen in Table 2.

Out of the total number of patients, 67 (69.07%) were negative and 30 (30.93%) positive for the Mantoux test at the initial evaluation. All 30 patients had treatment with MTX before starting biological treatment. None of the patients showed any active lesions suggestive for pulmonary MTB infection on chest X-ray. Patients with LTBI followed oral chemoprophylactic treatment for 9 months with isoniazid 300 mg/day (9 HIN), and the start of biological treatment was timed by 1 or 2 months.

The PASI score was evaluated before and after prophylactic treatment for LTBI (Table 3). Those who had a positive Mantoux test at the initial evaluation were excluded from this group. We observed an increase in the PASI score when Mantoux tested positive. (*p* = 0.022).

In order to obtain an optimal response to biologic therapy, 25 patients needed concomitant MTX and biological therapy. Of these, ten presented a positive IDR at the initial evaluation and 15 had a negative IDR. During the follow-up, ten patients with combined therapy were diagnosed with LTBI. Of the 72 patients in the group with monotherapy, 52 presented with a negative Mantoux test at initial evaluation, and 20 had a positive test. In this group of patients, 14 patients were diagnosed with LTBI during follow-up. The stratification of patients with LTBI according to the administered therapy is presented in Table 4.

We compared the group of patients with positive Mantoux test co-administered with MTX with biologic agents and the group with the Mantoux positive test in treatment with biologic agents only, excluding the patients who had a positive Mantoux test at the initial evaluation. We noticed that there is a higher frequency of positive Mantoux tests among patients with associated therapy (χ^2^ = 7.99; *p* = 0.0046) (Table 5).

During the study the occurrence of LTBI was monitored in relation to the biological treatment period. Thus, 30 patients (13 females and 17 males) were diagnosed with LTBI at the first evaluation (for the beginning of biologic therapy), seven patients after 1 year from initiation therapy, six after a period of 2 years of biologic therapy, and 11 patients later than 3 years after using the biologic agents, mean time 3.45. For the patients who had LTBI during the biological therapy (*n* = 24; 35.8%), ten (41.6%) were undergoing therapy with adalimumab, five (20.8%) with etanercept, six (25%) with infliximab, and one (4.1%) each with guselkumab, ustekinumab and ixekizumab.

Patients who were diagnosed with LTBI and had chemoprophylaxis [(total *n* = 54; 55.6%); (females *n* = 20; 37.03%; males *n* = 34; 62.96%)] were followed up for 1 year (±3 months) after the start of chemoprophylaxis. Eight patients had IDR > 15 mm one year after the LTBI diagnosis but with no signs of aTB on pulmonary X-ray. In these patients, microscopic examination of sputum (Ziehl-Neelsen stanning, optical microscopy with immersion lens) and GenExpert MTB test (a molecular diagnostic test capable of detecting MTB) were performed. All tests had negative results; therefore, the reaction was considered hyperreactive to PPD.

Since 2005, 31 (31.95%) patients were identified that had initiated biological therapy, and who by the end of the observation (December 2022), required a change of biological therapy. Of these, 21 followed prophylactic treatment with 9 HIN during the first biological treatment and none showed TB reactivation during the follow-up period (mean time = 3.28 years) after switching. The rest of the patients (*n* = 10) are among those who had negative Mantoux tests during the follow-up.

## 4. Discussion

It is important to approach psoriatic patients by examining their entire clinical context. This implies a thorough anamnesis which emphasizes their history and associated comorbidities. These details are important in order to corroborate the clinical form of psoriasis with triggers or exacerbations, and also to help guide therapeutic decision-making. In a review published in January 2023, the authors evaluated patients who had more frequent comorbidities associated with psoriasis vulgaris and the effectiveness and safety of biological treatments. Thus, in psoriatic arthritis, anti TNF-α molecules, secukinumab, ixekizumab, guselkumab and risankizumab are indicated as a first-line treatment. For patients with obesity, anti-IL17 drugs are recommended for their efficiency and for the possibility of dosing these drugs based on weight. Adalimumab and infliximab are the first therapeutic option in inflammatory bowel diseases. Adalimumab is also a suitable option for cardiovascular diseases as well as in depression, while in heart failure, anti-IL-12/23 and anti-IL-17 are preferred. In psoriasis associated with malignant diseases, anti-IL-17 and anti-IL-23 seem to be safe [8].

The infectious risks given by biological drugs are another important topic that we must consider when prescribing treatment. In psoriasis, antimicrobial peptides are more expressed. It plays a role in stimulating toll-like receptors that produce interferon (INF)-α and INF-β, promoting the activation and migration of myeloid dendritic cells in the lymph nodes and the secretion of IL-12 and IL-23 [9]. The latter modulate the activation of Th1 and Th17. These cellular and cytokine processes have an important role in protecting against infections. Biological anti-psoriatic agents target at different levels in this immunological cascade, and while they have favorable effects on skin lesions, may increase the risk of infections. One of the most important bacillary infections that can be triggered by biological therapy is LTBI.

Patients with LTBI are a reservoir of infection, and the risk of progression to aTB is still an uncertainty. The detection of patients with LTBI and treatment to prevent the appearance of the aTB is an important component for reducing infections and active diseases [7]. In the BCG vaccinated population, tuberculin skin test (TST) activates both the adaptive and the innate immune response. It produces the recall response to the PPD of MTB, thus, specific and functional responses of CD8+ and CD4 T-cells occur, producing INF-γ activation of macrophages and the production of TNF and IL-1 [10]. Patients with biological treatment belong to the category at risk of TBI, especially those with anti-TNF-α treatment [11,12,13]. Although the incidence of TB cases has decreased in the last few years in the European Union, Romania has reported a quarter of all TB cases in 2019 [14,15], which produces a higher risk of disease for patients with biological therapy [16,17].

At the same time, it is established that MTX raises the risk of TB [18,19,20]. As many of the patients with moderate and severe psoriasis were treated with MTX before starting biological therapy, it cannot be known for sure which of the drugs induced the appearance of LTBI. MTX, as an immunosuppressant of T lymphocytes, should produce false-negative Mantoux tests. MTX has a cytotoxic effect on cell lines with rapid proliferation [21], and likely produces oxidative stress which can initiate apoptosis [22] Vanessa Lucília Silveira de Medeiros et al. conducted a study in which they evaluated MTX’s relationship with TSTs in patients with psoriasis, from an endemic area, with a BCG vaccinated population at birth. The Mantoux test was performed and measured at the beginning and after 12 weeks of treatment with MTX, and positive tests have increased after MTX therapy. The evaluators also tested the serum INF-γ levels before and after the MTX treatment, observing high values after therapy [23]. In the BCG vaccinated population after birth, the reaction to TST may be false positive in the first 3 years of life. A study conducted on the healthy pediatric population and without immunosuppressive treatment showed that after 3 years of life the BCG vaccine should no longer be considered as a causal factor for IDR greater than or equal to 10 mm [24].

Human peripheral blood mononuclear cells when studied in vitro show greater expression of inflammatory cytokines (TNFα, Igγ, IL-6, and IL-1β) 24 h after being exposed to 10 µM of MTX [25]. Another prospective single-center study shows that the T-cells of the treated MTX patients produce more INF-γ than the untreated ones [26]. Moreover, after apoptosis induced by MTX of cells in psoriasis plaques, it was discovered in a new test with PPD that the cells had a greater ability to contain the protein [23]. All these could be causes of false positive Mantoux tests in MTX treated psoriatic patients. Although some of the patients treated with MTX are diagnosed with LTBI, a very small number of them have a conversion to aTB [27]. Cases of cavitary lung TB [28], spinal TB [29] may occur after low doses of MTX. In countries in which TB is endemic, LTBI therapy is instituted in patients at high risk of reactivation of LTBI. This could be a reason why the cases of reactivation of LTBI are limited. According to the national guide for TB prevention and treatment, LTBI treatment in immunosuppressed adult patients consists of 300 mg isoniazid/day for 9–12 months, without any other therapeutic alternative. In the United States of America, there are three therapeutic alternatives: three months of once-weekly isoniazid plus rifapentine (3 HP), four months of daily rifampin (4 R), three months of daily isoniazid plus rifampin (3 HR), or six/nine months of daily or twice-weekly of isoniazid monotherapy [30]. Short-term therapy (3 HP, 4 R, 3 HP) is preferred, with the same safety and efficiency as the 9 HIN treatment, but with a lower toxicity, safety, and high treatment compliance rates [31].

To exclude the diagnosis of LTBI induced by MTX, we observed how much time after the initiation of biological therapy Mantoux tests were positive in patients receiving monotherapy with a biological agent. The mean time from the beginning of biological therapy to the appearance of LTBI was 3.45 years without any correlation between the average treatment period and the risk of LTBI.

In accordance with our results, various other authors have noted that anti TNF-α treatments lead to a greater risk of TB reactivation [12,32,33]. Patients with concomitant therapy (biological treatment + MTX) had positive Mantoux tests with a higher frequency than those treated only with biologicals, and 80% of them combined an anti TNF-α with MTX. TNF-α is an important cytokine for tuberculous granuloma formation and maintenance. It has a synergistic effect with INF-γ and activates macrophages, stimulates apoptosis, ensures the migration to the infection site of monocytes and antigen specific T-cells, and activates cytotoxic T-cells [34,35]. This could be a reason for the higher number of LTBI patients among those treated with combined therapy (MTX + anti TNF-α) [36]. The role of IL-23 and IL-17 in the immune response to tuberculous infection is shown in multiple experimental murine studies, however it seems that their role is to regulate inflammation rather than protection against tuberculous infection [37,38]. Thus, it is possible that inhibitory therapy of these ILs has a lower aTB or LTBI rate in comparison to TNF-a inhibitors.

There is currently no protocol for choosing biological treatments guided by the particularities of the patients, but recommendations for choosing biological agents according to co-morbidities and patients’ risks are beginning to appear. A review published by Akshitha Thatiparthi et al. suggests IL-17 inhibitors or IL-23 inhibitors should be used in patients with LTBI, with ustekinumab or TNF-α inhibitors after TB prophylaxis [39]. Comparative studies between a wide range of non-TNF-α inhibitors are needed to conclude which biological is best suited for each individual patient.

In our study, the PASI score was negatively influenced, due to being increased when the Mantoux test was positive. However, the prophylactic treatment for LTBI has no impact on the effectiveness of biological therapy in patients with moderately severe psoriasis according to a study published by V. Neamțiu et al. [40]. In spite of that, the worsening of the PASI score can be explained by the activation of various immune mechanisms during the occurrence of infections [41]. In one case report, the authors note the improvement of psoriasis after LTBI treatment in a 58 years old woman. Remission was maintained for 1 year after the completion of the anti-tuberculous treatment [42]. In a review published in 2021 that looked at how infections can influence the evolution of psoriasis, the curative treatment of streptococcal pharyngeal infections by tonsillectomy in selected cases of guttate psoriasis reduced the severity of psoriasis [43].

Although the risk of developing aTB or LTBI during biological treatment persists, the question arises of overdiagnosis by false positive Mantoux tests. In a study published by Bassukas ID et al., Mantoux tests of patients with moderate to severe psoriasis were compared with those with inflammatory bowel diseases before initiating a biological treatment. In patients with moderate to severe psoriasis, there is an overestimation of LTBI [44]. This would be explained by the intense skin inflammatory reaction of patients with moderate to severe psoriasis compared to others [45,46,47]. Although none of the patients in our study showed the reactivation of TB after oral prophylactic treatment with 9 HIN, several studies claim that this treatment does not give full protection [47,48,49], and the risk of aTB or LTBI is maintained during the biological treatment, especially with anti-TNF-α. The frequency of aTB and LTBI during IL blockers appears to be lower, and may be a better therapeutic option for patients at the highest risk [12,35,50,51].

## 5. Limitations

The study has a few limitations: we used a small group of patients, especially for anti-IL drugs; a lack of analyses with higher sensitivity and specificity for the detection of TBI, e.g., INF-γ release assays (the National Therapeutic Protocol for psoriasis treatment requires as a screening test for TBI only the Mantoux test as a screening test for TBI and the medical system does not financially support the performance of this type of test).

## 6. Conclusions

It is still necessary to carry out screening for TBI in patients with moderate to severe psoriasis planned for biological therapy intervention, especially in countries with high infection rates. Romania has a high epidemic risk of TBI, and it is the country with the most cases of multi drug resistance TB (MDR TB) in the European Union [14], so we consider that it is necessary to maintain a severe follow-up protocol for patients with biological therapy, even if in our study there were no cases of TB reactivation. However, tests with higher specificity such as IFN-γ release assays would be required to reduce LTBI overdiagnosis. At the same time, conducting comparative studies between a wider range of biological therapies and their safety for TBI (depending on the patients’ risks) could help doctors in choosing individualized biological therapy.

## Figures and Tables

**Table 1:** Comorbidities of the patients in biological therapy.

Comorbidities	%
Psoriatic Arthritis	41.2%
Cardiovascular diseases	
○ Arterial hypertension	38.1%
○ Ischemic heart disease	14.4%
○ Heart failure	3%
Anxiety/depression	6.1%
Diabetes mellitus	11.3%
Dyslipidemia	42.2%
Obesity	19.5%
Non-alcoholic steatohepatitis	31.9%
Inflammatory bowel disease	3%
Chronic Obstructive Pulmonary Disease	6.1%

**Table 2:** Infections that occurred during the administration of biological therapy.

Types of Infections	Sex	Mean Age	Number of Patients
Gastrointestinal infection	B	53.1	18
	F	63.5	13
Pharyngeal infections	B	57.1	6
	F	58.7	6
Urinary infections	B	50.8	6
	F	58.7	22
Candidiasis	B	57.5	4
	F	53.5	4
LTBI	B	58.8	15
	F	64.7	9
Pneumonia	B	57	2
	F	75	1
Viral hepatitis type B	B	58.6	3
	F	66	2
Viral hepatitis type C	B	-	0
	F	69	2

LTBI = latent tuberculosis infection.

**Table 3:** PASI before LTBI treatment compared to PASI after 9 HIN treatment.

	Mean	Median	Min	Max	SD	SE
PASI score before LTBI treatment	6.95	4	0	35	7.59	1.55
PASI score after LTBI treatment	3.12	2	0	15	3.62	0.74
*t*-test = 0.022						

PASI = Psoriasis Area and Severity Index; LTBI = latent tuberculosis infection; 9 HIN = 9 months of isoniazid 300 mg/day.

**Table 4:** Frequency of patients with LTBI depending on the therapy administered.

Biologic Therapy	Monotherapy	LTBI Patients with Biological Monotherapy	Associated MTX to Biological Therapy	LTBI Patients with Biological Concomitant Therapy
Adalimumab	27	9	7	1
Etanercept	17	3	7	2
Infliximab	11	1	6	5
Ixekizumab	3	0	2	1
Secukinumab	2	0	0	0
Guselkumab	2	0	1	1
Risankizumab	3	0	0	0
Ustekinumab	7	1	2	0
Total	72	14	25	10

LTBI = latent tuberculosis infection; MTX = methotrexate.

**Table 5:** Comparison between the frequency of patients diagnosed with LTBI treated only with biological therapy and those treated with MTX associated with biological drugs.

	Positive Mantoux Test (LTBI)	Negative Mantoux Test	Total
Concomitant administration therapy (MTX and biologic agents)	10	5	15
Single therapy (biologic agents)	14	38	52
Total	24	43	67
χ^2^ = 7.99			
*p* = 0.0046			

LTBI = latent tuberculosis infection; MTX = methotrexate.

## Data Availability

Data are available from the corresponding author (doriana.chilom@umft.ro).
